# STAT3, a Master Regulator of Anti-Tumor Immune Response

**DOI:** 10.3390/cancers11091280

**Published:** 2019-08-30

**Authors:** Cédric Rébé, François Ghiringhelli

**Affiliations:** Platform of Transfer in Cancer Biology, Centre Georges François Leclerc, INSERM LNC UMR1231, University of Bourgogne Franche-Comté, F-21000 Dijon, France

**Keywords:** STAT3, cancer, CD4^+^ T cells, CD8^+^ T cells, myeloid cells, immune check point

## Abstract

Immune cells in the tumor microenvironment regulate cancer growth. Thus cancer progression is dependent on the activation or repression of transcription programs involved in the proliferation/activation of lymphoid and myeloid cells. One of the main transcription factors involved in many of these pathways is the signal transducer and activator of transcription 3 (STAT3). In this review we will focus on the role of STAT3 and its regulation, e.g., by phosphorylation or acetylation in immune cells and how it might impact immune cell function and tumor progression. Moreover, we will review the ability of STAT3 to regulate checkpoint inhibitors.

## 1. Introduction

STAT3 is part of the Signal Transducer and Activator of Transcription (STAT) family which includes seven members encoded by distinct genes. STAT3 has evolutionary conserved amino acid sequences between *H. sapiens* and *S. harrisii* (99.09%) [1]. In resting cells, STAT3 remains in an inactive form in the cytoplasm. Once activated, mainly through phosphorylation, STAT3 translocates to the nucleus to play its transcription activity for specific target genes [2]. STAT3 phosphorylation on tyrosine (Y705) is mainly regulated by members of Janus-activated kinases (JAK), whereas its phosphorylation on serine (S727) is commonly regulated by mitogen-activated protein kinases, CDK5 and protein kinase C [3]. Finally, histone acetyltransferase-mediated reversible acetylation of STAT3 on a single lysine residue (K685) is a third mechanism of STAT3 activation through STAT3 dimer stabilization [4]. However, the phosphorylation on S727 is responsible for a mitochondrial relocalization of STAT3 where it exerts non-transcriptional roles. This mitochondrial localization enables STAT3 to increase cell respiration (through electron transport chain complex activation) and Ras transformation [5]. Non-nuclear STAT3 can also regulate glycolysis, thus enhancing lactate production leading to the protection of cells from apoptosis and senescence and can also regulate calcium homeostasis, energy production and apoptosis at the endoplasmic reticulum level [6].

Regulation of STAT protein activation is controlled by negative regulators, e.g., PIAS (protein inhibitor of activated STAT) and SOCS (suppressors of cytokine signaling) proteins as well as protein tyrosine phosphatases. PIAS are nuclear factors that negatively regulate STAT transcriptional activity through many mechanisms, especially by interacting and thus blocking the DNA binding activity [7]. SOCS proteins directly or indirectly interact with tyrosine kinase SH2 domains to prevent JAK from activating STAT3 [8]. Protein tyrosine phosphatases (such as CD45, SHP-1 and SHP-2) remove phosphates from activated STATs, which represent a third level of STAT modulation [9,10,11]. Lastly, STAT3 has also been shown to go through ubiquitination-dependent proteosomal degradation [12]. Moreover, because of their homologies, STATs can form homodimer and heterodimers. Specificity depends on the activator signal and leads to the transcription of different target genes. For example, STAT3 can heterodimerized with STAT1, under IL-6 treatment [13].

It is now well-established that STAT3 signaling is a major intrinsic pathway driving apoptosis, inflammation, cellular transformation, survival, proliferation, invasion, angiogenesis and metastasis in cancer [14,15,16,17]. Moreover, STAT3 in cancer cells affects stromal cells function, establishing crosstalk between cancer cells and its microenvironment. For example STAT3 can dampen STAT1-mediated upregulation of MHC class I, allowing immune escape [1]. The other way for STAT3 to drive tumor immune escape is to regulate the function of stromal cells and more particularly immune cells. 

In general, all seven STAT family members have prominent roles in T-cell function or T-cell differentiation, survival or expansion. STAT4 is essential for Th1 and STAT6 is important for Th2 differentiation. Similarly, all STAT proteins have all seven prominent roles in myeloid cells and they all influence each other’s expression and activity status on complex and not understood chromatin regulation. All that makes the interpretation of complex immune cell scenarios triggered by multiple action of cytokines, growth factors, hormones and chemokines a tricky business to correctly relate functions to this or that STAT family member. Importantly, T-cell expansion by common γ-chain cytokines and many T-cell effector functions such as CD8^+^ T-cell, γδ T-cell generations and cytokine release function and mounting a killing or efficient cytokine signaling response against foreign or mutated antigen is a STAT5-mediated affair together with proper recognition and signaling through the T-cell receptor (TCR), where again interplays are not carefully understood or worked out [18,19]. Furthermore, STAT5 is also essential to generate Treg cells, where both *Foxp3* and *Cd25* are direct STAT5 target genes [20]. STAT5 has also essential functions in erythropoiesis or macrophage or dendritic cell (DC) polarization, but due to space constrains and focus on fine-tuning and twisting immune responses in health or disease we will here illuminate STAT3 function in immune cells. We illuminate many important immune modulatory interplays of STAT3 signaling in distinct T-cell and myeloid cell compartments. We describe current knowledge on the impact of STAT3 activation in immune cells on the balance between immunosurveillance and immunoescape. We will describe how STAT3 affects both myeloid and lymphoid cells usually in a way to inhibit anti-tumor immune response and to promote tumor growth.

## 2. STAT3 and T-Cells

T lymphocytes or T-cells play a central role in host adaptive immune response to cancer [21]. Tumor-infiltrating CD4^+^ and CD8^+^ T-cells are associated with varying clinical outcomes and survival in many types of cancer such as colorectal, [22] breast [23] and lung cancers [24]. Cytokines can shape T-cells immune response and tune CD4^+^ T-cells differentiation and CD8^+^ T-cells activation [25]. Among T-cells, different subsets have been described (regulatory T-cells, cytotoxic T-cells, T helper cells) with distinct functions that could be regulated by STAT3 (Table 1). 

### 2.1. Th1/Th2

CD4^+^ T helper (Th) cells assist other hematopoietic cells in immune processes, including activation of cytotoxic T lymphocytes (CTLs), natural killer (NK) cells and macrophages. Th cells are activated when stimulated with a peptidic sequence of the antigen they specifically recognize. These peptides are presented to Th cells by antigen presenting cells (APCs), such as DCs through MHC class II molecules. Once activated, they rapidly proliferate and secrete cytokines that will inhibit or assist the active immune response [26].

Expression levels of Th1 cell genes coding for Interferon-γ (IFNG), TAP1, Granzym B (GZMB) are significantly higher in colorectal tumors than in normal tissue. A high expression of Th1 cytotoxic genes was associated with significantly improved disease-free survival whereas a low expression of those genes lead to disease recurrence [63]. T-BET, the master transcriptional regulator for Th1 differentiation is induced by TCRs and IL-12 stimulation [64]. In contrast, GATA-3 is the master assessor for Th2 differentiation, after stimulation with IL-4 [65]. IL-27, a member of the IL-6/IL-12 family produced by macrophages and DCs, favors Th1 differentiation by up-regulating T-BET, down-regulating GATA-3 and suppressing proinflammatory cytokine production such as IL-2, IL-4, and IL-13 [27,66]. In this context, only the IL-27-dependent Th1 proliferation was mediated by STAT3 [27]. In contrast, in a different cytokine context, in patients harboring STAT3 dominant negative mutations or STAT1 or IL-21R loss of function mutations, it was shown that IL-21/IL-21R, STAT3 and STAT1 signaling are required for in vitro differentiation of IL-10-secreting cells, related to Th2 [28].

### 2.2. Th17

Th17 cells are CD4^+^ T-cells induced by TCR triggering together with IL-6, transforming growth factor (TGF)-β, and IL-23, an IL-12 family member, stimulation [67]. Th17 cells have emerged as key drivers of a wide range of autoimmune disorders, including inflammatory bowel disease, psoriasis, and ankylosing spondylitis [68]. Th17 cell expansion was observed in human cancers such as ovarian, melanoma, breast or colon cancers [69]. In colorectal cancer, patients with low expression of Th17 genes seem to have a prolonged disease-free survival [63]. However, a positive role of Th17 was proposed in melanoma and ovarian cancer. Therefore, the role of Th17 cells in cancer immunity remains controversial [70].

Th17 are characterized by the expression of the transcription factors RORγt and RORα and the production of IL-17A [71]. In addition, STAT3 is also essential for Th17 cell differentiation, since STAT3 ablation in mice CD4^+^ T-cells, abrogates Th17 differentiation [29,30]. Moreover, in patients harboring STAT3 dominant negative mutations, IL-21R loss of function mutations or STAT1 gain of function mutations, it was shown that IL-21/IL-21R/STAT3 signaling is required for in vitro production of IL-17A/F by Th17 cells whereas STAT1 overexpression inhibits it [28]. STAT3 can associate with Trim28 and RORγt to drive the transcription of target cytokines such as IL-17A/F [72]. Moreover, many in vitro studies have shown that STAT3 can be activated by several pro-inflammatory cytokines including IL-6, IL-21 and IL-23 leading to the regulation of RORγt and RORα expression, along with the development and the stabilization of Th17 cells [31,32,33,34,35]. We found that the n-3 fatty acid docosahexaenoic acid (DHA) was able to induce the expression of SOCS3 in a PPARγ-dependent manner. SOCS3 then inhibits pSTAT3 and Th17 differentiation [36]. Another regulator of STAT3 activation in Th17 is the tyrosine phosphatase SHP1, which dampens IL-6- and IL-21-driven Th17 development and limits colitis in mice [37]. Similarly, the Platelet Factor 4 (PF4) can also up-regulate SOCS3 expression leading to the inhibition of STAT3, Th17 differentiation and tumor growth [73].

The STAT3 acetylation profile is also involved in Th17 polarization. First, lysyl oxidase-like 3 (LOXL3) is able to deacetylate STAT3 and to inhibit its transcriptional activity. *Loxl3* deficiency leads to constitutive STAT3 K685 acetylation causing reduced Th17 differentiation associated with resistance to DSS-induced colitis in mice [38]. Second, we showed that metformin and resveratrol, two SIRT1 activators, entail STAT3 acetylation leading to Th17 differentiation impairment and limit tumor growth in mice. The capacity of metformin to promote acetylation of STAT3 and to decrease Th17 differentiation was also shown in patients [39]. 

We have shown that in vitro Th17 cells differentiated with IL-6 and TGF-β and in vivo tumor-infiltrating Th17 cells express CD39 and CD73 ectonucleotidases. This ectonucleotidase catalytic machinery entails the degradation of extracellular ATP into adenosine, an immunosuppressive molecule which suppresses effector T-cells. The expression of ectonucleotidases is dependent on IL-6-driven STAT3 activation and TGF-β-mediated downregulation of the zinc finger protein Growth Factor Independent-1 (Gfi-1), both required for the transcriptional regulation of ectonucleotidase expression during Th17 cell differentiation. CD39 expression at the surface of Th17 cells fosters tumor growth, suggesting that the immunosuppressive functions of Th17 cells in cancer are dictated by ectonucleotidase expression [40]. It has been reported that naive T-cells can be differentiated into Th17 cells with IL-1β, IL-6, IL-23 and without TGF-β. Unlike Th17 cells generated with TGF-β and IL-6, these Th17 cells were highly pathogenic in vivo [74] and didn’t express ectonucleotidases [40]. These observations propose STAT3 and Gfi-1 as key determinants in the immunoregulatory function of Th17 cells, at least in part through the regulation of ectonucleotidase expression [40].

The mechanistic role of STAT3 in Th17 positive effects on anti-tumoral response is less documented. TAM-derived exosomes can deliver miR-29a-3p and miR-21-5p to CD4^+^ T-cells leading to the inhibition of STAT3 and consequently to Th17 polarization in favor to Treg, which is beneficial for epithelial ovarian cancer progression [41].

### 2.3. Treg

Suppressor regulatory T-cells (Treg) maintain peripheral immune tolerance [75,76]. Co-stimulation of naive CD4^+^ T-cells with TCR and TGF-β, triggers the generation of CD4^+^/CD25^+^ Treg cells. This leads to the expression of FOXP3, Tregs master transcription factor [77]. These T-cells accumulate in tumors and in cancer patients peripheral blood [78]. An increase in Treg frequency is generally considered as a marker of poor prognosis in cancer, probably because Treg mediate suppression of anti-tumor immunity [79,80,81].

The role of STAT3 in *Foxp3* expression regulation in Tregs appears to be context-dependent. In vitro, IL-2 induces the binding of STAT3 and STAT5 to a highly conserved STAT-binding site located within the first intron of the *Foxp3* gene, leading to FOXP3 expression up-regulation in purified CD4^+^CD25^+^ T-cells but not in CD4^+^CD25^−^ cells [42].

In tumor-infiltrating Tregs, both STAT3 and STAT5 bind to a STAT consensus site in the *Foxp3* promoter to enhance FOXP3 expression which seems to be important in maintaining Tregs inhibitory functions [42,43,44]. S1PR1 (Sphingosine-1 Phosphate Receptor 1) signaling has been shown to restrain Treg number and functions. An increase in S1PR1 in CD4^+^ T-cells promotes STAT3 activation and JAK/STAT3-dependent Treg tumor migration, whereas STAT3 ablation in T-cells diminishes tumor-associated Treg accumulation and tumor growth [45]. Treatment of metastatic cancer or chronic myelogenous leukemia after allogeneic hematopoietic stem cell transplantation in patients with low-dose IL-2, leads to an increase of peripheral blood CD4^+^CD25^+^ cells and to FOXP3 expression in CD3^+^ T-cells [42]. STAT3 and FOXP3 co-operatively control a subset of genes, responsible for Treg cell ability to suppress Th17 cell-mediated inflammation [82]. In contrast, IL-21 which activates STAT3 but not STAT5 has no effect on Treg viability, activation or function, suggesting that in this context IL-21-mediated STAT3 activation is not sufficient [83]. The regulation of *Foxp3* expression by STAT3 was strengthened by other studies. CDK5 increases *Foxp3* gene expression through phosphorylation of STAT3 at serine 727 [47]. GATA-3 controls the expression of miR-125a-5p, which in turn inhibits the expression of IL-6R and STAT3 and dampens Treg conversion [48]. In contrast, differentiation of naive T-cells into Tregs in vitro, is impaired when STAT3 is activated (by IL-6 or IL-27) [34,46]. In a different context, STAT3 binds to a silencer element within the *Foxp3* locus [84] and could also inhibit STAT5 interacting with the *Foxp3* promoter [85], to prevent FOXP3 expression. 

One possible explanation for these ambivalent actions of STAT3 on Treg differentiation could be the phosphorylation site. Indeed, wogonin, a natural flavonoid from *Scutellaria baicalensis,* inhibits Treg induction by down-regulating ERK and STAT3-Y705 phosphorylation and promoting NF-κB and STAT3-S727 activation [49]. The modulation of STAT3 activity by molecular compounds could lead to inhibition of Tregs activity. Thus, WP1066 (an inhibitor of STAT3 signaling) enhances T-cell cytotoxicity against melanoma through inhibition of FOXP3^+^ Tregs [50]. Compound9#, a fluorinated β-amino-ketone molecule, also inhibits Treg induction both in vitro and in vivo, via blockage of JAK2 signaling [51]. Finally, STAT3 inhibition with anti-sense oligonucleotides in association with radiation is a potent therapeutic target against Tregs [52]. All these studies suggest that STAT3 is required for immunosuppressive functions of Tregs. 

### 2.4. T Follicular Helper Cells 

Tfh differentiation is complex because it requires interaction with other cells such as B cells or DC. In mice, IL-6, IL-21 and IL-27 are essential for Tfh formation while in humans, Tfh generation relies on TGF-β, IL-12, IL-23. In both mammalian species of rodents or humans, Tfh cells express BCL6, ASCL2, IL-21, PD-1, and ICOS and produce IL-21 and CXCL13 [86,87]. While the role of Tfh is ambiguous in lymphoid tumors, many studies report that accumulation of Tfh in tertiary structures within the tumor is of good prognosis for breast, colon and non-small cell lung cancer patients [88,89]. Even if the protective effects of Tfh cells seem to be dependent on IL-21 and CXCL13-mediated recruitment of leucocytes, little is known about the accurate mechanism of Tfh anti-tumoral effects. 

Generation of Tfh cells in patients with impaired STAT3 DNA-binding function is compromised due to the inability of IL-12 to induce IL-21 production without affecting its capacity to induce ICOS, BCL6 or CXCR5 expression [54]. When siRNA specific for STAT3 was used, TGF-β, IL-12, IL-23 failed to induce BCL6 expression in vitro [53]. However, the requirement of STAT3 seems to depend on the differentiation status of Tfh cells: It is required for Tfh generation but once these cells are generated it is no longer required [90]. In the same context, murine STAT3-deficient CD4^+^ T-cells, Tfh cells expressed less or more BCL6 and IL-21 according to the immune environment [91,92]. Moreover, in conditions where STAT3 is necessary for Tfh differentiation, two studies showed that it cooperates with the IkZF transcription factors Aiolos and Ikaros. Moreover, the kinase activity of ROCK2 (Rho-associated coiled-coil kinase 2, an actin cytoskeleton assembly regulator) is required to induce STAT3 phosphorylation, nuclear relocalization and DNA binding to regulate *Bcl6* expression [93,94]. Finally, the importance of STAT3 in Tfh differentiation was strengthened by its capacity to block the expression of the Th2-associated genes *Gata3* and *Il4* [53,55].

A new protumorigenic IL-21^+^ Tfh-like cell subset with a CXCR5^−^PD-1^−^ BTLA^−^CD69^hi^ phenotype was identified in hepatocellular carcinoma (HCC). STAT1 and STAT3 activation are critical for these Tfh-like cell induction which operate via IL-21-IFN-γ pathways to induce plasma cells and create conditions for M2b macrophage polarization and tumor growth [56].

The importance of STAT3 in Tfh differentiation and its pro- or anti-tumoral role is not clear and could be dependent on differentiation stage, localization and environment.

### 2.5. Th9

Th9 cells have been characterized as a proinflammatory CD4 T-cell subset that can be generated through TGF-β and IL-4 stimulation. These cells are characterized by IL-9 secretion. Th9 harbor potent IL-9-dependent anti-cancer properties in most solid tumors and especially in melanoma while they can promote the development of many hematological human tumors, including Hodgkin’s lymphoma and other B cell lymphoma. Th9 cells activate both innate and adaptive immune responses, thereby favoring anti-cancer immunity and tumor elimination [95]. 

In this CD4 T-cell subset, STAT3 was shown to dampen IL-9 production through STAT5 inhibition in Th9 cells [57]. In vitro, Th9 long term ability to secrete IL-9 is inhibited by pSTAT3 through an IL-10 receptor signaling [58]. In contrast, in humans Th9, pSTAT3 (mainly driven by IL-21 self-induction) inhibits pSTAT1-mediated T-BET induction, through SOCS3 induction. Since T-BET is an inhibitor of IL-9 transcription, this sustains IL-9 production. Patient-derived Th9 cells with dysfunctional STAT3, lose their capacity to produce IL-9, because of SOCS3 expression down-regulation, which leads to an increase pSTAT1 and T-BET expression. In the same study, the loss of function mutations observed in patients were recapitulated with deletion studies in mice, revealing that absence of STAT3 culminates into increased IL-9 production [59].

### 2.6. CD8^+^

CD8^+^ T lymphocytes are central players in cancer immune response, through their capacity to kill malignant cells. Upon recognition by the TCR of specific antigenic peptides presented on the surface of target cells by human leukocyte antigen class I (HLA-I)/beta-2-microglobulin (β2m) complexes, the CTL effector functions are activated. These functions are mediated either directly, through exocytosis of cytotoxic granules containing perforin and granzym into the target cells, resulting in cancer cell destruction, or indirectly, through secretion of cytokines, including IFN-γ and TNF [96].

The stimulation of the human and murine CD8^+^ T-cells CD28, stabilizes the tyrosine kinase Lck activity and pSTAT3-mediated transcription of NKG2D. NKG2D expressing CD8^+^ T-cells exert cytolytic activity against target tumor cells in vitro and significantly improve the antitumor therapeutic effects in vivo [62]. Even if IL-10 is considered as an immune suppressor it can also increase expansion and cytotoxic activity of CD8^+^ cells. Tumor-resident CD8^+^ T-cells express high levels of IL-10R, leading to high levels of activated pSTAT3 and pSTAT1 in response to IL-10 [97]. In contrast, circulating CD8^+^ T-cells from peripheral blood of HCC patients present high amounts of pSTAT3 which is correlated with high amount of IL-4, IL-6 and IL-10 and low quantity of IFN-γ which may result in abnormal immune surveillance against tumor cells [98]. In murine CD8^+^ T-cells, STAT3 has been shown to inhibit both IFN-γ-mediated CXCL10 production by myeloid cells and CD8^+^ T-cells CXCR3 expression (the receptor of CXCL10), blocking the migration of these cells to the tumor site [60].

In an adoptive transfer therapeutic strategy in mice, ablating STAT3 in CD8^+^ T-cells prior to transfer, allows efficient tumor infiltration and robust CD8^+^ T-cell proliferation, resulting in increased tumor antigen-specific T-cell activity and tumor growth inhibition [61].

Altogether STAT3 seems to inhibit CD8^+^ T-cell expansion and cytolytic activity except in some particular conditions.

## 3. STAT3 and Myeloid Cells

APCs dictate immune system response, since these cells have been shown to capture antigens in the periphery, migrate to the lymphoid organs, and present processed peptides to T-cells in a way that may lead either to priming or tolerance induction [99]. Among myeloid cells, different subsets have been described (Macrophages, DCs, Myeloid-Derived Suppressor Cells (MDSCs)) with distinct functions that could be regulated by STAT3 (Table 2).

### 3.1. Macrophages

Tumor-associated macrophages (TAMs) are subdivided into two subsets, M1 and M2 macrophages, based on their capacity to express or produce Nitric Oxide Synthase/IL-12/TNF-α or arginase-1/IL-10/TGF-β, respectively. M1 has potent microbicidal properties and promotes Th1 responses, whereas M2 supports Th2-associated effector functions [143,144]. M2 macrophages includes M2a, M2b or M2c subtypes. Tumor-derived signals, such as Macrophage-Colony Stimulating Factor (M-CSF/CSF-1), Monocyte Chemoattractant Protein-1 (MCP-1), or Chemokine (C-C motif) Ligand-2 (CCL2) entails the accumulation of M2 at the tumor site. M2 macrophages participate in tumor growth by releasing proangiogenic cytokines and growth factors, e.g. Epidermal Growth Factor (EGF), Vascular Endothelial Growth Factor (VEGF), Platelet-Derived Growth Factor (PDGF), Colony Stimulating Factor-1 (CSF-1), and basic Fibroblast Growth Factor (βFGF). They also generate arginase-1, IL-10 and TGF-β. These molecular messengers will inhibit the antitumor function of T-cells and NK cells. This will favor tumor tolerance and impairment of antitumor immunotherapies efficacy [145,146,147].

IL-6 inhibition of M-CSF-induced colony formation observed in animals was abolished in mice mutated for the gp130-STAT1/3 signaling, suggesting that the IL-6/STAT3 pathway could regulate macrophage homeostasis [148]. Moreover, breast cancer-derived exosomes are capable of inducing IL-6 secretion and a pro-tumoral phenotype (IL-6, IL-10, CCL2 production) in macrophages, partially *via* gp130/STAT3 signaling [106]. More particularly, STAT3 directly induces the expression of the M2 marker CD163, both in macrophages and tumor cells [103]. Prostate-cancer cells induce a change in macrophage phenotype from M1 into M2, through STAT3 activation [104]. This can be induced by Plasminogen Activating Inhibitor (PAI)-1 secreted by cancer cells [105].

The inhibition of STAT3 signaling in either macrophages or bone marrow-derived DCs is of great importance in cancer immunotherapy, because it allows these APCs to restore the responsiveness of tolerant T-cells from tumor-bearing mice. STAT3 signaling is a negative regulator in peritoneal elicited macrophages, as its targeted disruption gives a constitutively activated phenotype and an increased ability to produce inflammatory mediators in response to LPS. This may be the consequence of an increased STAT1 activity (leading to high production of inflammatory factors) or a lack of IL-10 production [100]. Moreover, macrophages derived from conditional STAT3 knockout mice are better than wild-type macrophages to prime cognate CTL responses and to cross-present tumor-derived antigen to CTLs in vitro. This leads to a more important proliferation of CTLs and an increased production of IFN-γ and TNF-α. Similarly, removing STAT3 in hematopoietic cells, leads to rapid activation of innate immunity by CpG (a TLR9 ligand), with enhanced activation of macrophages, neutrophils and NK cells and production of IFN-γ, TNF-α, IL-12 to eradicate B16 melanoma tumors [102]. Targeting STAT3 signaling therefore represents an attractive strategy to increase CTL responses in the tumor-bearing host [101]. Immunosuppressive activities of TAMs correlate with over-activated STAT3 signaling, whereas disruption of TAMs STAT3 activity can enhance rat immune response to breast cancer [149]. In glioblastoma patients, tumor-infiltrating macrophages were shown to be predominantly STAT3-positive M2 macrophages, which are associated with a poor prognosis [150]. The same team proposed that corosolic acid and oleanic acid can prevent tumor formation through their capacity to suppress macrophages M2 polarization and tumor cell proliferation by inhibiting STAT3 activation [107,108]. CD163-targeted corosolic acid-containing liposomes were also shown to reprogram M2 macrophages into M1 (increased expression of TNF-α, IFN-γ, IL-12, IL-2 and decreased expression of IL-10) [109]. Since ERK5 mediates Y705 phosphorylation of STAT3 in myeloid cells, blocking ERK5 might constitute a treatment strategy to reprogram macrophages toward an antitumor state by inhibiting STAT3-induced gene expression [110]. In intrahepatic cholangiocarcinoma (ICC), patients with high counts of CD163^+^ M2 macrophages showed poor disease-free survival. Tumor cell supernatant from HuCCT1 ICC cell lines induces the production of IL-10 and VEGF-A by macrophages through activation of STAT3 and polarization towards the M2 phenotype [113]. Similarly, renal cell carcinoma-derived BMP (Bone Morphogenetic Protein)-6 mediates IL-10 expression in macrophages through Smad5 and STAT3, and M2 polarization [114]. These observations were confirmed by the fact that macrophages isolated from mouse tumors displayed activated STAT3 and induced angiogenesis in an in vitro tube formation assay via STAT3 induction of angiogenic factors, including VEGF and βFGF [115]. STAT3 signaling within the tumor microenvironment induces the production of IL-23, a procarcinogenic cytokine, via direct transcriptional activation of the IL-23/p19 gene in TAMs, while inhibiting the production of IL-12, a central anticarcinogenic cytokine, thereby shifting the balance of tumor immunity towards carcinogenesis [44]. The M-CSF-inducible DC-SIGN (Dendritic cell-specific ICAM-3-grabbing nonintegrin or CD209) expression along monocyte-to-macrophage differentiation is dependent on JNK and STAT3 activation. DC-SIGN contributes to the release of IL-10 that would maintain STAT3 activation in tumor cells, thus implying that DC-SIGN favors the maintenance of an activated STAT3 context in the tumor stroma. This will compromise the ability of TAMs and DCs to generate an effective antitumor response and to maintain an immunosuppressive environment [111]. This effect is potentiated by STAT3-activating cytokines IL-6 and IL-10 produced by STAT3-activated tumor cells. In the same way, IL-6-derived from gastric cancer cells induces normal macrophages differentiation to M2 macrophages with higher IL-10 and TGF-β expression, and lower IL-12 expression, via STAT3 phosphorylation. IL-6-induced M2 macrophages exert a pro-tumor function by promoting GC cell proliferation and migration [151].

However, an anti-tumoral role of STAT3 in macrophages has also been proposed, based on studies that investigated the importance of STAT3 in macrophages through an indirect manner, using SOCS3 conditional knockout mice in macrophages. Hyperactivation of STAT3 in myeloid cells simultaneously exerted anti-inflammatory as well as anti-tumor effects [112]. Thus, Lipoxin A4 induces STAT3 phosphorylation and mediates differentiation of monocytic-like cells into M2 subtypes with anti-tumorigenic activities [152]. The discrepancies between these studies could be explained by the fact that SOCS3 and lipoxin signaling should regulate other pathways such as NF-κB.

### 3.2. Dendritic Cells (DCs)

DCs are another differentiated stage of monocytes and are the key APCs of the immune system. DCs play a main role as immune sentinels in the initiation of T-cell response against microbial pathogens, tumors and inflammation [153,154]. 

The first evidence that STAT3 is important for DCs fate was made in mice lacking STAT3 expression in hematopoietic progenitors. These animals present a profound deficiency in DCs which are unresponsive to Flt3L stimulation [116]. However, the same mice bearing a tumor, present enhanced DC, T-cell, NK cell and neutrophil functions and a decreased tumor progression [117]. DCs derived from LysM^cre^/STAT3^flox/flox^ mice display higher cytokine production in response to TLR stimulation and activate more efficiently T-cells. Intratumoral administration of these DCs significantly inhibits MC38 tumor growth [119]. Moreover, ablating STAT3 in myeloid cells increases CpG-induced DCs maturation, T-cell activation, tumor antigen-specific T-cells generation and long-lasting antitumor immunity in B16 melanoma tumor model [102]. Similarly, CpG was used to administer STAT3 siRNA specifically to myeloid and B cells. Ablation of STAT3 in these cells increases DC engagement and adoptively transferred CD8^+^ T-cells effector functions in vivo, with an upregulation of effector molecules such as perforin, granzym B, and IFN-γ [118]. Mice without STAT3 in myeloid compartment of tumor stroma, including DCs and macrophages, present reduced numbers of tumor-infiltrating CD4^+^CD25^+^/FOXP3^+^/LAG3^+^ Tregs, along with an increase in CD8^+^ effector T-cells [117]. Constitutive STAT3 activation in tumor-residing DCs reduces expression of MHC class II and costimulatory molecules, impairs the antigen-presenting function of DC and contributes to the expansion of tumor-infiltrating FOXP3^+^ T-cells and attenuates CD4^+^ Th cell responses [117,122,123]. This can be partly explained by the fact that STAT3 inhibits the ID2 (inhibitor of differentiation 2) expression which promotes tumor immunity [124]. Finally, IL-6 is a suppressor of bone marrow-derived DC activation/maturation and a regulator of DC differentiation in vivo, through STAT3 phosphorylation. Then, DC differentiation/maturation is controlled by IL-6-gp130-STAT3, suggesting that this amplification loop may represent a target for controlling T-cell-mediated immune responses [155]. Similarly, mammary tumor-derived exosomes inhibit the differentiation of murine myeloid precursor cells into DCs in vitro. This was correlated with an increased IL-6 level and phosphorylated STAT3 and was blocked in bone marrow cells derived from IL-6 knockout mice. This suggests that tumor cells can dampen DCs differentiation through an autocrine activation of STAT3 by IL-6 [125]. 

In humans, tumor-derived factors suppress DC generation through STAT3-mediated PKCβII reduced expression [156]. STAT3-depleted DCs with adenoviral STAT3 short hairpin RNA (shRNA) or siRNA presented an altered cytokines production profile under TLR stimulation (such as more IL-12 and TNF-α and less IL-10), and induced tumor Ag-specific T-cells and IFN-γ-producing γδ T lymphocytes more efficiently than control DCs [119,120,121]. The impact on IL-12 can be explained by a competition of STAT3 with CDK9 on a binding site on the IL-12p35 promoter [157]. The effects of STAT3 on IL-10 expression can be explained by the fact that HDAC6 forms a complex with STAT3, recruited to a specific DNA sequence element in the *Il10* gene promoter [158]. 

### 3.3. Myeloid-Derived Suppressor Cells (MDSCs)

MDSCs have been identified in humans and mice as a population of immature myeloid cells with the ability to suppress T-cell activation [159]. In tumor-bearing mice, these cells have been shown to markedly expand in lymphoid organs and blood [160]. In addition, an increased MDSCs frequency was detected in the blood of patients with different types of cancers [161,162]. In mice and humans, MDSCs from tumor bearers induce antigen-specific MHC class I restricted tolerance of CD8^+^ T-cells and are one of the major suppressors of antitumor immunity [163]. In humans the phenotype of MDSCs is a matter of debate. However, two major subsets of MDSCs have been identified: monocytic MDSCs (M-MDSCs), similar to monocytes and polymorphonuclear MDSCs (PMN-MDSCs) sharing phenotypic and morphologic features with neutrophils [164].

STAT3 is probably one of the main transcription factors that regulate MDSCs functions. MDSCs from tumor-bearing mice present high levels of phosphorylated STAT3, compared with immature myeloid cells from naive mice [128]. Moreover, ablation of STAT3 expression through the use of conditional knockout mice or selective STAT3 inhibitors (JSI-124) markedly reduce the expansion of MDSCs, promote accumulation of DCs and increase T-cell responses in tumor-bearing mice [117,127,128]. In mice, when STAT3 was specifically deleted in myeloid cells, the ability of MDSCs to inhibit CD4^+^ and CD8^+^ T-cell-dependent apoptosis by cell-cell contact and to induce Treg polarization through TGF-β, IL-10 and NOX2 secretion was decreased [131]. When STAT3 was specifically turned off in myeloid cells expressing TLR9 from prostate cancer patients, using a CpG-STAT3 siRNA or CpG-STAT3 antisense oligonucleotides, it abrogated the immunosuppressive effects of patient-derived MDSCs on effector CD8^+^ T-cells [129,130]. In contrast, deletion of SOCS3 in myeloid cells leads to an increased activation of the STAT3 and to differentiation into Gr-1^+^CD11b^+^Ly6G^+^ MDSCs, enhancing tumor growth [165]. STAT3 can also be regulated in MDSCs by the epigenetic-associated protein, p66a, which may physically interact with STAT3 to suppress its activity through posttranslational modification [166]. We have shown that tumor-derived exosome (TDE)-associated HSP70 led to STAT3 activation in MDSCs. This activation depends on TLR2/MyD88 and autocrine production of IL-6. TDEs from human tumor cell lines activate human MDSCs suppressive functions but not their expansion in an HSP70/TLR2-dependent manner, showing that the mechanism described in mice is also relevant in cancer patients [133]. In tumor cell supernatants, tumor soluble factors induce activation of ERK, which results in MDSCs expansion, while TDEs trigger STAT3 activation without promoting MDSCs expansion [133]. How can these discrepancies be explained? It is well known that STAT3 signaling in myeloid cells, entails the expression of Bcl-xL, c-myc, cyclin D1 or survivin, which favors cell proliferation and inhibits cell apoptosis and differentiation to mature cell types [15,167]. 

STAT3 controls the G-CSF-and G-MCSF responsive induction of C/EPBβ (CCAAT-enhancer-binding protein β) expression and the interferon regulatory factor 8 (IRF8) downregulation in myeloid cells [126,134]. The transcription factor C/EBPβ was reported to play a crucial role in controlling the differentiation of myeloid precursors to functional MDSCs [126] whereas IRF8 attenuated MDSC accumulation, phenotype and function [134]. These data suggest also a link between CSF and STAT3 pathway in the regulation of MDSC biology.

Finally, recent studies highlighted the importance of signaling pathways downstream STAT3 in MDSCs differentiation. MDSCs isolated from mouse tumors present activated STAT3. STAT3 favors the production of angiogenic factors, including VEGF and βFGF to induce angiogenesis in an in vitro tube formation assay [115]. STAT3 as well as STAT5 control the cytokine-induced expression of the cytoplasmic NADPH oxidase NOX2 [168], being e.g., crucial for DNA damage response in AML cells, leading together with mitochondrial ROS production, which is a predominant STAT3 affair to the production of ROS, responsible for MDSCs-induced immune suppression in murine colon, lung, mammary carcinoma, thymoma, sarcoma models and in patients with head and neck cancers [132]. STAT3 also favors immunosuppressive functions of MDSCs by inducing the expression and the activity of IDO (Indoleamine 2,3-dioxygenase 1) in breast [137] and liver [135] cancers or arginase-1 in head and neck squamous cell carcinoma [136]. STAT3-inducible up-regulation of the myeloid-related protein S100A9 enhances MDSCs production in cancer. Mice lacking this protein mount potent antitumor immune responses and reject implanted tumors, an effect reversed by administration of wild-type MDSCs [169].

However, STAT3 activity can be endogenously controlled in intra-tumoral MDSCs. Thus, tumor hypoxia led to CD45 protein tyrosine phosphatases activation, resulting in STAT3 activity downregulation and in M-MDSC differentiation into tumor-promoting TAMs [138].

Moreover, many inhibitors were proposed such as the XIAP inhibitor embelin [139], PM01183 a novel synthetic agent derived from trabectedin [140], alisertib (MLN8237), a small-molecule inhibitor of Auror-A kinase [141], STATTIC or BBI608 [142] to inhibit STAT3 in MDSCs with a potential clinical application to favor anti-tumor response.

## 4. STAT3 and Check Points Inhibitors

The emergence of immune ‘checkpoint inhibitors’, blocking negative regulators of T-cell immunity opened new clinical applications of cancer immunotherapies. The more widely studied are cytotoxic T lymphocyte antigen 4 (CTLA-4) and programmed death receptor 1 (PD-1). CTLA-4 is mainly expressed on T helper and Treg cells and binds to its ligands B7–1 (CD80) and B7–2 (CD86) on APCs [170]. PD-1 expression is induced both on activated CD8^+^ T-cells, Tfh and Treg present in tumor microenvironment, and on activated B cells and NK cells. PD-1 has two ligands: PD-L1 (B7-H1) and PD-L2 (B7-DC). PD-1 signaling contributes to T-cell exhaustion [170]. Other checkpoints can also be implicated in tumor immune escape such as Lymphocyte activation gene 3 protein (LAG-3) and T-cell immunoglobulin and mucin domain-containing 3 (TIM-3), IDO1, B- and T-lymphocyte attenuator (BTLA), V-domain immunoglobulin suppressor of T-cell activation (VISTA) and the A2A adenosine receptor (A2A-R) [170,171].

STAT3 can bind the promoter of murine *Pdcd1* gene coding for PD-1 in T-cells [172]. STAT3 has been demonstrated to bind to the *Pdcd1l1* promoter (coding for PD-L1) to regulate its transcriptional expression in cancer cells. It requires either mutated oncogene chimeric nucleophosmin/anaplastic lymphoma kinase (ALK) in T-cell lymphoma [173], Latent membrane protein-1 (LMP1) of Epstein–Barr virus in nasopharyngeal carcinoma [174], HDAC6 in osteosarcoma [175], NF-κB in melanoma [176] or HIF-1α in pulmonary adenocarcinoma [177]. In contrast, a study shows that STAT3 is necessary for docetaxel-mediated inhibition of PD-1 expression [178].

In CRC patients, FGFR2 expression is correlated to PD-L1 expression. FGFR2 stimulation leads to STAT3 activation, PD-L1 expression and colon cancer cell death [179]. The EGFR signaling pathway can also regulate PD-L1 expression through the IL-6/JAK/STAT3 pathway in non-small cell lung cancer (NSCLC) cells [180,181]. The expression of PD-L1 is associated with a poor prognosis and inhibition of PD-L1 expression (e.g., through STAT3 or its partner inhibition) is associated to a decrease in cell proliferation and/or an increase in tumor cell death in most of these studies. However, the effects on T-cell anti-tumor response were not tested here. 

PD-L1/L2 can be expressed in non-tumoral cells. In liver MDSCs, GM-CSF is responsible for STAT3 activation which in turn induces PD-L1 and IDO1 expression [135]. GBM cells secrete IL-6 which in turn activates STAT3 in infiltrated myeloid cells leading to STAT3 activation and PD-L1 expression. An anti-IL-6 therapy decreases PD-L1 expression and tumor size in a CD8^+^ T-cell-dependent manner [182]. In HCC, a similar regulation was observed. In this setting, the HCC-associated fibroblasts produce IL-6 which in turn increases PD-L1 expression in neutrophils [183]. Similarly, in prostate carcinoma, DC generated in the presence of stromal myofibroblasts factors expressed significantly elevated levels of PD-L1 in a STAT3 and IL-6-dependent manner [184]. In chronic lymphocytic leukemia (CLL), STAT3 is required to PD-L1 expression and IL-10 production which in turn seems to be responsible for PD-1 expression in CD4^+^ and CD8^+^ T-cells [185]. In adult T-cell lymphoma, IL-27B production by Lymphoma cells and IL-27p28 production by macrophages lead to STAT3 activation and PD-L1/L2 expression in macrophages [186]. TGF-β is another cytokine secreted by inflammatory or tumor cells that can increase PD-L1 expression in DC in a STAT3-dependent manner [187]. The use of IFN-α in clinical oncology for many types of cancers is reconsidered, as IFN-α induces the expression of PD-L1 molecule, in the majority of the specific immune cell populations, particularly in DCs [188]. However, it should be noted that interferons are highly liver toxic and they act on all cell types in the body, where many unwanted side effects from neurotoxic problems to fever symptoms were reported in therapy, making their window of opportunity in fragile patients delicate.

Little is known about the ability of STAT3 to regulate other checkpoints. One study shows that STAT3/IRF1 are required for PD-L2 expression in melanoma cells [189]. In a non-cancerous context, it has been shown that CTLA-4 as well as PD-1, LAG-3 and TIM-3 expression is induced in HIV-infected DCs in a STAT3-dependent manner [190]. 

In contrast, the immunosuppressive effect of these checkpoints can be dependent on STAT3 activation in target-cells. For example, IDO1 may promote EMT (Epithelial-Mesenchymal Transition) by activation of the IL-6/STAT3/PD-L1 signaling pathway [191]. TIM-3^+^ endothelial cells modulate T-cell response to lymphoma surrogate antigens by suppressing activation of CD4^+^ T lymphocytes through the activation of the IL-6-STAT3 pathway, inhibiting Th1 polarization and providing protective immunity [192]. Finally, CTLA-4 critically shapes the characteristics of IL-17 producing CD8^+^ cells (Tc17 a pro-tumoral population) in a STAT3 activation-dependent manner and inhibition of CTLA-4-induced STAT3 activity reverses Tc17 signature to Tc1-like cells with enhanced cytotoxic potential [193].

## 5. Conclusions

STAT3 regulates genes involved in biological functions of cancer and immune cells, rendering this pathway an interesting therapeutic target. STAT3 could be inhibited directly by peptides or natural compounds or indirectly by blocking upstream signaling pathways such as IL-6 and JAK2 pathways (For review see [194,195]). However, the complexity of STAT3 biology and its broad effects may render its clinical development complex. This review underlines the ambivalent effects of STAT3 on the antitumoral immune response, with both positive or negative effects, depending on the context or cell types involved. Additional translational studies on patients treated with STAT3 inhibitors are awaited to understand their effects on the complexity of tumor biology.

## Figures and Tables

**Table 1:** Impact of STAT3 in T-cell subsets.

Immune Cell Family	STAT3 Role in	STAT3 Modulators	Effects	Reference
**CD4^+^ T-cells**	Th1	IL-27	↗ proliferation	[27]
	Th2	IL-21	IL-10 secretion	[28]
	Th17	KO STAT3	Inhibition of Differentiation	[29,30]
		IL-6, IL-21, IL-23	↗ RORγt and RORα → Differentiation (↗ IL-17)	[31,32,33,34,35]
		PPARγ ligand, Platelet factor 4	↗ SOCS3 → Inhibition of Differentiation	[36]
		SHP1	↘ pSTAT3 → Inhibition of Differentiation	[37]
		LOXL3	↘ STAT3 deacetylation → Differentiation	[38]
		Metformin, Resveratrol	SIRT1 activation → STAT3 acetylation → Th17 Differentiation	[39]
		IL-6 (+ TGF-β)	↗ STAT3 activation and ↘ Gfi-1 → ↗ CD39 and CD73 expression	[40]
		miR29a-3p, miR-21-5p	↘ STAT3 → Th17 Differentiation	[41]
	Treg	KO STAT3	↘ number of Treg	
		IL-2	↗ STAT3 + STAT5 → ↗ FOXP3 → ↗ inhibitory functions	[42,43,44]
		S1PR1	↗ STAT3 → Treg migration in the tumor	[45]
		IL-6, IL-27	↗ STAT3 → ↘ FOXP3 → ↘ Treg differentiation	[34,46]
		CDK5	↗ pSTAT3(S727) → ↗FOXP3	[47]
		GATA-3	↗ miR125a-5 → ↘ IL-6R + STAT3 → ↘ Treg conversion	[48]
		Wogonin	↘ pSTAT3(Y705) ↗ pSTAT3(S727) → ↘ Treg differentiation	[49]
		WP1066	↘ pSTAT3 → ↘ FOXP3^+^ Treg	[50]
		compound9# (fluorinated β-amino-ketone)	↘ FOXP3^+^ Treg	[51]
		anti- sense oligos + radiations	↘ FOXP3^+^ Treg	[52]
	Tfh	KO STAT3 or STAT3 siRNA	↘ BCL6 ↗ GATA-3, IL-4 (Th2)	[53]
		STAT DNA Binding domain mutation	↘ STAT3 activity → ↘ IL-21	[54]
		TGF-β	↗ pSTAT3 → ↘ GATA-3, IL-4 (Th2)	[53,55]
		Intratumoral Tfh-like cells	→ ↗ IL-21, IFN-γ → ↗ M2b	[56]
	Th9	KO STAT3	↗ pSTAT5 → ↗ IL-9	[57]
		Murine IL-10	↗ pSTAT3 → ↘ IL-9	[58]
		Human IL-21	↗ pSTAT3 → ↘ T-BET → ↗ IL-9	[59]
**CD8^+^ T-cells**		KO STAT3	↗ IFN-γ → ↗ CXCL10 production by myeloid cells	[60]
			↗ CXCR3 (CXCL10 receptor)	
			↗ CD8^+^ proliferation and tumor invasion	[61]
		CD28 stimulation	↗ pSTAT3 → ↗ NKG2D	[62]

**Table 2:** Impact of STAT3 in myeloid cells.

Immune Cell Family	STAT3 Role in	STAT3 Modulators	Effects	Reference
**yeloid cells**	Macrophages	KO STAT3	Inflammatory macrophages ↗ CTL activation ↗ TLR9 pathway (IFN-γ, TNF-α, IL-12) ↗ IL-23 and ↘ IL-12	[100] [101] [102]
		STAT3 oxerexpression	↗ CD163 (M2 marker)	[103]
		cancer cells (PAI-1, BMP6, IL-6)	↗ pSTAT3 → polarization of M1 into M2 (CD163)	[103,104,105]
		Tumor exosomes	↗ pSTAT3 → ↗ IL-6, IL-10, CCL2	[106]
		Corosolic acid, oleanic acid	↘ STAT3 activity → M1 polarization (↗ TNF-α, IFN-γ, IL-12, IL-2)	[107,108,109]
		ERK5	↗ pSTAT3 → pro-tumor macrophages	[110]
		M-CSF	↗ pSTAT3 → ↗ DC-SIGN	[111]
		KO SOCS3	↗ pSTAT3 → anti-tumor macrophages	[112]
		Tumor cells	↗ pSTAT3 → ↗ IL-10, VEGF, βFGF → angiogenesis	[113,114,115]
	Dendritic Cells	KO STAT3 or siRNA or shRNA	↗ DCs APC function and cytokine production (↗ IL-12, TNF-α and ↘ IL-10)	[102,116,117,118,119,120,121]
		Constitutive STAT3 activation	↘ MHC class II, ID-2	[117,122,123,124]
		Tumor-derived exosomes and IL-6	↘ PKCβII → DCs activation and differentiation	[125]
	MDSCs	KO STAT3 or siRNA or anti-sense oligonucleotides	↘ C/EBPβ ↘ immunosuppressive functions → MDSCs differentiation into DCs ↘ NOX2 → ↘ ROS → ↘ immunosuppression	[126] [117,127,128,129,130] [131,132]
		Tumor-derived exosomes (HSP70)	↗ pSTAT3 → ↗ immunosuppressive functions (with an IL-6 amplification loop)	[133]
		CCL2	↗ pSTAT3 → PMN-MDSCs-mediated T-cell suppression	
		IL-6	Early-stage MDSCs accumulation	
		G-CSF	↗ pSTAT3 → ↘ IRF8	[126,134]
		GM-CSF	↗ pSTAT3 → ↗ IDO → ↗ immunosuppression	[135]
		siRNA or STATTIC	↘ pSTAT3 → ↘ arginase-1 → ↘ immunosuppression	[136]
		JSI-124 (STAT3 inhibitor)	↘ pSTAT3 → ↘ IDO → ↘ immunosuppression	[137]
		CD45	↘ pSTAT3 → M-MDSCs differentiation into TAMs	[138]
		Embelin, PM01183, alisertib, STATTIC or BBI608	↘ pSTAT3	[139,140,141,142]
		Tumor cells	↗ pSTAT3 → ↗VEGF, βFGF → ↗ angiogenesis	[115]
